# Data-Driven Clinical Phenotyping of Adult Epilepsy Using Latent Class Analysis: A Regional Cohort Study from Southern Kazakhstan

**DOI:** 10.3390/jpm16070344

**Published:** 2026-06-25

**Authors:** Nurlybek Mombekov, Nigara Yerkhojayeva, Aliya Ualiyeva, Nazira Zharkinbekova, Cigdem Ozkara, Gulnaz Nuskabayeva, Karlygash Sadykova, Assylbek Mombek, Bakhytkul Yernazarova, Tangsholpan Zholdassova, Rissalat Abdullayeva, Aziz Nabiyev, Nursultan Nurdinov

**Affiliations:** 1 Department of Special Clinical Disciplines, Faculty of Medicine, Khoja Akhmet Yassawi International Kazakh-Turkish University, Turkestan 161200, Kazakhstan; nurlybek.mombekov@ayu.edu.kz (N.M.); nuskabayeva.gulnaz@ayu.edu.kz (G.N.); karlygash.sadykova@ayu.edu.kz (K.S.); 2 Department Epidemiology, Biostatistics and Evidence-Based Medicine, Farabi University, Almaty 050000, Kazakhstan; aliya.ualiyeva@kaznu.kz; 3 Department of Neurology, Psychiatry, Rehabilitation and Neurosurgery, South Kazakhstan Medical Academy, Shymkent 160019, Kazakhstan; nazirazhar@mail.ru; 4 Department of Neurology, Cerrahpasa Medical Faculty, Istanbul University, Istanbul 34342, Turkey; cigdemoz@iuc.edu.tr; 5 Department of Fundamental Medical Sciences, Faculty of Dentistry, Khoja Akhmet Yassawi International Kazakh-Turkish University, Turkestan 161200, Kazakhstan; assylbek.mombek@ayu.edu.kz; 6 Department of Surgery and Pediatric Dentistry, Faculty of Dentistry, Khoja Akhmet Yassawi International Kazakh-Turkish University, Turkestan 161200, Kazakhstan; bakhytkul.yernazarova@ayu.edu.kz (B.Y.); tangsholpan.zholdassova@ayu.edu.kz (T.Z.); rissalat.abdullayeva@ayu.edu.kz (R.A.); 7 Department of Therapeutic and Orthopedic Dentistry, Faculty of Dentistry, Khoja Akhmet Yassawi International Kazakh-Turkish University, Turkestan 161200, Kazakhstan; nabiev.aziz@ayu.edu.kz

**Keywords:** adult epilepsy, latent class analysis, clinical phenotyping, drug-resistant epilepsy, clinical heterogeneity, quality of life, cognition, regional cohort

## Abstract

**Background/Objectives:** Adult epilepsy is clinically heterogeneous, and individual clinical predictors may not fully capture the multidimensional burden associated with drug-resistant epilepsy (DRE). This study aimed to identify latent clinical phenotypes in adults with epilepsy and examine their cross-sectional associations with DRE and broader disease burden. **Methods:** This regional observational cohort study used a source database of 1100 patients with epilepsy. After excluding two patients aged <18 years, the adult analytic cohort included 1098 patients. Complete-case latent class analysis (LCA) was performed in 1054 patients using age at onset, disease duration, seizure type, seizure frequency, serial seizures/status, postictal confusion, neurological status, neuroimaging category, and number of antiseizure medications. Model selection was based on statistical fit, class size, and clinical interpretability. Internal clinical validation outcomes included DRE, quality of life, cognitive screening, and stigma scores. Post hoc characterization described the classes by epilepsy etiology, derived epilepsy type, and seizure categories aligned with current terminology. **Results:** A three-class solution was selected, with class sizes of 314, 465, and 275. DRE prevalence increased stepwise across classes: 5.7%, 14.2%, and 33.1%, respectively (*p* < 0.001). In adjusted analysis, Class 2 had higher odds of DRE than Class 1 (odds ratio 2.70, 95% confidence interval 1.56–4.67), while Class 3 showed the strongest association (odds ratio 8.19, 95% confidence interval 4.15–16.16; both *p* < 0.001). Higher-burden classes showed lower quality-of-life and cognitive scores and higher stigma scores. **Conclusions:** LCA identified three clinically interpretable, burden-enriched phenotypic profiles associated with a stepwise gradient in DRE and broader multidimensional disease burden. These cross-sectional profiles may provide a useful framework for describing clinical heterogeneity in adult epilepsy and generating hypotheses for future validation studies.

## 1. Introduction

Epilepsy is one of the most common serious neurological disorders worldwide and remains a major source of disability, psychosocial burden, and healthcare utilization [1,2]. In adults, epilepsy is clinically heterogeneous and encompasses a wide range of etiologies, seizure types, comorbidities, treatment responses, and long-term functional outcomes [3,4].

Drug-resistant epilepsy (DRE) is defined as failure of two tolerated and appropriately selected antiseizure medication regimens to achieve sustained seizure freedom [5]. Long-term cohort studies show that the likelihood of durable seizure freedom decreases after repeated medication failure [6]. Factors associated with DRE include earlier age at onset, longer disease duration, structural brain abnormalities, higher seizure burden, neurological deficits, and more complex treatment exposure [7,8,9,10].

Adult epilepsy is not only a disorder of seizure recurrence. It is a multidimensional condition that affects quality of life, cognition, psychosocial functioning, stigma, and adherence to treatment. The Quality of Life in Epilepsy Inventory-31 (QOLIE-31) is widely used to assess epilepsy-specific health-related quality of life and has been applied across diverse populations [11,12]. Cognitive impairment is also clinically relevant in adult epilepsy, and screening instruments such as the Montreal Cognitive Assessment (MoCA) are increasingly used to identify cognitive difficulties in clinical and research settings [13]. Beyond seizure control, cognitive dysfunction, psychosocial burden, perceived stigma, and medication adherence are important determinants of disease impact and functional outcome in people with epilepsy [14,15,16,17,18,19].

These considerations support the use of multidimensional phenotyping rather than reliance on individual clinical predictors. Conventional analyses often examine clinical variables separately and may not capture how seizure burden, neurological status, imaging findings, psychosocial factors, cognitive status, and treatment exposure cluster within individual patients. Latent class analysis (LCA) is a probabilistic, data-driven method used to identify unobserved subgroups within heterogeneous populations based on patterns of co-occurring characteristics [20]. This approach may be useful when clinical heterogeneity cannot be adequately explained by single predictors alone, although careful model selection, class interpretation, and validation are essential [21].

In epilepsy research, data-driven phenotyping has been used to refine clinical classification and characterize patient subgroups beyond traditional diagnostic categories. Quantitative phenotypic approaches can add value to conventional electroclinical classification in familial epilepsies [22], and latent profile approaches have identified clinically meaningful cognitive phenotypes in temporal lobe epilepsy [23,24]. However, such approaches remain underused in regional adult epilepsy cohorts, particularly in Central Asia.

This gap is especially relevant for Kazakhstan and neighboring regions, where adult epilepsy datasets with detailed clinical, cognitive, psychosocial, and treatment-related variables remain limited. Broader reviews of epilepsy in Asian countries emphasize variation in epidemiology, access to care, diagnostic capacity, and treatment pathways across the region [25]. Kazakhstan-specific data have demonstrated important national trends in epilepsy using electronic health system records, but such studies are primarily epidemiological and do not fully address multidimensional clinical phenotyping [26]. Recent Central Asian analyses based on global burden estimates further highlight the need for region-specific evidence on epilepsy burden and care pathways [27]. At the same time, studies from low- and middle-income settings emphasize persistent gaps in specialist access, continuity of care, and structured epilepsy management, reinforcing the need for practical approaches to identify patients with higher clinical burden [28,29].

Therefore, this study aimed to identify latent clinical phenotypic profiles in adults with epilepsy using LCA and to examine their cross-sectional associations with DRE and multidimensional disease burden in a regional cohort from Southern Kazakhstan.

## 2. Materials and Methods

### 2.1. Study Design and Setting

This was a regional observational cohort study with a cross-sectional analytical framework. The source database included 1100 patients with epilepsy identified from regional epilepsy registries and neurological care settings in Southern Kazakhstan, including the Turkestan and Kyzylorda regions. The clinical data used for the present study were generated during routine clinical care and patient assessment and were collected between 1 November 2025 and 30 December 2025, after approval by the Local Ethics Committee. Data extraction, organization, and analysis for the present manuscript were subsequently performed from January to March 2026.

**Figure 1:**
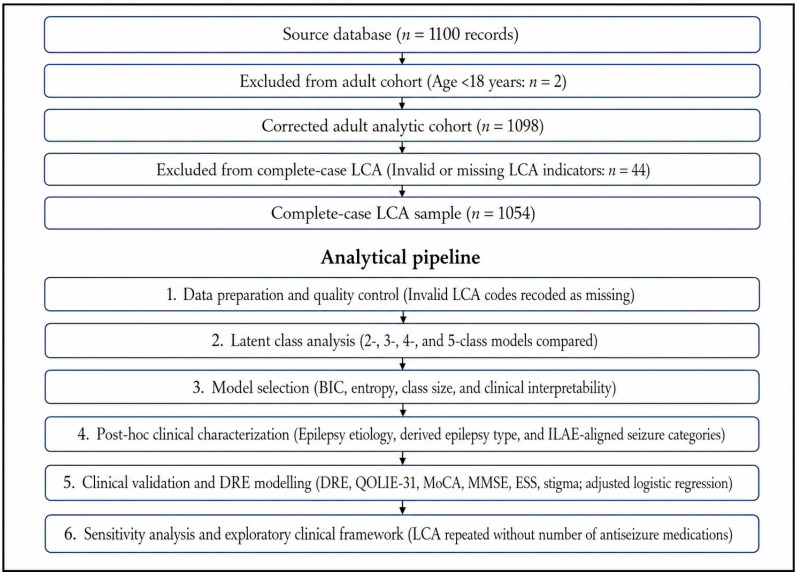
Study flowchart and analytical pipeline. LCA, latent class analysis; BIC, Bayesian information criterion; DRE, drug-resistant epilepsy; QOLIE-31, Quality of Life in Epilepsy Inventory-31; MoCA, Montreal Cognitive Assessment; MMSE, Mini-Mental State Examination; ESS, Epilepsy Stigma Scale; ILAE, International League Against Epilepsy.

Data were obtained from medical records and structured patient interviews. During data quality control, two patients aged <18 years were excluded, resulting in a corrected adult analytic cohort of 1098 patients. For latent class analysis, complete-case analysis was applied to the predefined core LCA indicators. Accordingly, 44 records were excluded because at least one core LCA indicator was missing, unknown, not examined, or invalidly coded. The final complete-case LCA sample included 1054 patients (Figure 1). Variable-specific missingness across the predefined core LCA indicators is summarized in Supplementary Table S1.

The present study used the same regional source cohort as a previously reported analysis of clinical and patient-reported outcomes in adult epilepsy [30]. However, the present analysis addresses a distinct research question. Specifically, it applies LCA to identify data-driven clinical phenotypes and evaluates their association with DRE and multidimensional disease burden. Thus, the current study is methodologically and conceptually distinct from prior descriptive or outcome-specific analyses of the same cohort.

### 2.2. Eligibility Criteria

Eligible participants were adults aged 18–75 years with a definite clinical diagnosis of epilepsy, disease duration of at least two years, current follow-up or treatment by a neurologist, and available clinical documentation sufficient for seizure and treatment characterization. Patients were excluded if they were younger than 18 years, had inadequate seizure documentation, required urgent hospital care for non-seizure systemic conditions, had severe clinical deterioration precluding reliable assessment, or had a history of alcohol or substance abuse. Patients with missing, unknown, not examined, or invalid values in any predefined core LCA indicator were excluded only from the complete-case LCA analysis, as described below.

### 2.3. Data Sources and Clinical Variables

Clinical and sociodemographic data were obtained from patients’ medical records and structured interviews. Sociodemographic variables included age, sex, ethnicity, marital status, educational level, occupation, and place of residence. Clinical variables included age at epilepsy onset, disease duration, seizure type, seizure frequency, seizure timing, serial seizures or status epilepticus, number of antiseizure medications, family history of seizures, postictal confusion, neurological status, comorbid conditions, and neuroimaging findings. CT/MRI findings were categorized as normal, abnormal, or not examined.

### 2.4. Variables Used for Latent Class Analysis

The final predefined core LCA indicator set included nine variables: age at epilepsy onset, disease duration, seizure type, seizure frequency, serial seizures/status epilepticus, postictal confusion, neurological status, CT/MRI category, and number of antiseizure medications. These variables were selected before model fitting because they reflected key dimensions of epilepsy burden, including temporal disease characteristics, seizure phenotype, seizure severity, neurological involvement, structural imaging status, and treatment exposure.

Non-informative codes, including “unknown,” “not examined,” and invalid category values, were recoded as missing rather than treated as clinically meaningful categories. Complete-case LCA was restricted to these predefined core indicators only; therefore, patients with missing, unknown, not examined, or invalidly coded values in any of these indicators were excluded from the LCA sample. Video EEG was not included as a core LCA indicator because the code “0” denoted “not examined” rather than a normal video EEG result. Any VEEG-related summaries were used only for descriptive or post hoc clinical characterization and were not used to define latent class membership.

Because the number of antiseizure medications may be conceptually related to DRE status, we performed a sensitivity analysis excluding medication count from the LCA indicator set. Agreement between the primary and sensitivity class solutions was assessed using the adjusted Rand index. This analysis was used to evaluate whether the identified phenotypic classes were driven primarily by treatment exposure or reflected broader clinical patterns.

### 2.5. Outcomes Used for Internal Clinical Validation

The latent classes were evaluated using internal clinical validation outcomes that were not included as core LCA indicators. These outcomes were derived from the same regional cohort and, therefore, were not considered external validation in an independent dataset. They included DRE, quality of life, cognitive screening scores, and stigma. Quality of life was assessed using QOLIE-31, with total scores ranging from 0 to 100. Cognitive function was assessed using the MoCA, and global cognitive status was additionally summarized using the Mini-Mental State Examination (MMSE). Stigma was assessed using the Epilepsy Stigma Scale (ESS).

### 2.6. Definition of Drug-Resistant Epilepsy

DRE was defined according to the International League Against Epilepsy consensus definition as failure of adequate trials of two tolerated, appropriately chosen, and appropriately used antiseizure medication regimens, whether as monotherapy or in combination, to achieve sustained seizure freedom. In the present database, DRE status was operationalized using documented treatment history and seizure control status. Patients were classified as having DRE if medical records indicated persistent seizures despite at least two adequately selected and tolerated antiseizure medication regimens. Patients who achieved sustained seizure freedom or had not yet received two adequate medication trials were classified as non-DRE. DRE status was not used as a core LCA indicator and was evaluated only as an internal clinical validation outcome.

### 2.7. Model Selection and Class Assignment

Candidate latent class models with two to five classes were compared. The final model was selected by balancing statistical fit indices, convergence stability, minimum class size, and clinical interpretability. Patients were assigned to the class for which they had the highest posterior probability of membership.

### 2.8. Post Hoc Clinical Characterization of Latent Classes

After the final three-class LCA solution was selected, a post hoc clinical characterization was performed to improve the clinical interpretability of the data-driven phenotypes. The final patient-level class assignments were merged with registry-based seizure and etiology variables. Seizure categories were harmonized using ILAE-aligned terminology: registry-coded simple partial seizures were recoded as focal aware seizures, complex partial seizures as focal impaired awareness seizures, and secondary generalized seizures as focal to bilateral tonic–clonic seizures. Registry-coded generalized tonic–clonic seizures were reported as generalized/bilateral tonic–clonic seizures because detailed electroclinical syndrome-level confirmation was not available for all patients. Epilepsy etiology was summarized using broad categories and detailed registry-based categories. These post hoc variables were not used to define the latent classes. When available, VEEG variables were considered only as descriptive post hoc variables and were not included in the LCA model.

### 2.9. Statistical Analysis

Descriptive statistics were used to summarize the corrected adult analytic cohort and the complete-case LCA sample. Continuous variables were presented as mean with standard deviation or median with interquartile range, as appropriate. Categorical variables were summarized as frequencies and percentages.

Between-class comparisons for internal validation outcomes were performed using chi-square tests for categorical variables and non-parametric tests for continuous or ordinal variables. Statistical significance was defined as a two-sided *p*-value < 0.05.

LCA was performed in Stata version 16 using the generalized structural equation modeling framework with latent classes (gsem/lclass). Candidate models with two to five latent classes were estimated using maximum likelihood. Multiple starting values were used to reduce the risk of convergence to local maxima. Model selection was based on the Bayesian information criterion, adjusted Bayesian information criterion, entropy, class size, convergence stability, and clinical interpretability. The final model was selected by balancing statistical fit with stability and clinical plausibility. Patients were assigned to the class for which they had the highest posterior probability of membership. Average posterior probabilities for the final three-class solution were calculated to assess classification certainty.

Adjusted logistic regression was used to examine the association between latent class membership and DRE. Class 1 was used as the reference category. The model was adjusted for age, sex, disease duration, educational level, and place of residence. Results were reported as odds ratios with 95% confidence intervals. Model performance was assessed using the area under the receiver operating characteristic curve. Calibration was evaluated using the Hosmer–Lemeshow test, and multicollinearity was assessed using variance inflation factors.

A sensitivity analysis was performed by repeating LCA after excluding the number of antiseizure medications from the indicator set. Agreement between the sensitivity and primary class solutions was assessed using the adjusted Rand index. All analyses were conducted using Stata version 16.

### 2.10. Ethics

The study was approved by the Local Ethics Committee of Khoja Akhmet Yassawi International Kazakh-Turkish University, Turkestan, Kazakhstan (Protocol No. 45, 21 October 2025). Written informed consent was obtained from all participants. The study was conducted in accordance with ethical principles for research involving human participants.

## 3. Results

The source database included 1100 patients with epilepsy. After exclusion of two patients aged <18 years, the corrected adult analytic cohort comprised 1098 patients. Complete-case LCA was performed in 1054 patients after exclusion of 44 records with invalid or missing values in core LCA indicators.

The mean age of the corrected adult cohort was 40.45 years (SD 14.33), with a median age of 39 years (IQR 29–51). Female sex accounted for 557/1097 patients (50.8%), and urban residence was reported in 539/1098 patients (49.1%). Overall, DRE was present in 182/1097 patients (16.6%), and disability of any type was reported in 562/1097 patients (51.2%).

At the cohort level, the median QOLIE-31 score was 60 (IQR 45–70), the median MoCA score was 21 (IQR 18–23), the median MMSE score was 22 (IQR 19–25), the median ESS score was 23 (IQR 18–25), and the median stigma score was 40 (IQR 31–45). Baseline demographic and clinical characteristics of the corrected adult cohort are summarized in Table S2.

**LCA model selection.** After recoding invalid LCA indicator values as missing, the complete-case LCA included 1054 patients. Candidate models with two to five latent classes were compared. Among these models, the three-class solution was selected because it provided the best balance between statistical fit, entropy, adequate class size, convergence stability, and clinical interpretability.

For the selected three-class model, the log-likelihood was −10,023.313, AIC was 20,266.627, BIC was 20,812.265, adjusted BIC was 20,462.887, entropy was 0.791, and the minimum class size was 275. The class sizes in the selected model were 314, 465, and 275 patients. Although the four- and five-class models improved AIC, they did not improve BIC relative to the three-class solution and produced less clinically interpretable subdivisions. Therefore, additional model complexity was not considered justified on combined statistical and clinical grounds. Model fit indices are presented in Table S3.

**Figure 2:**
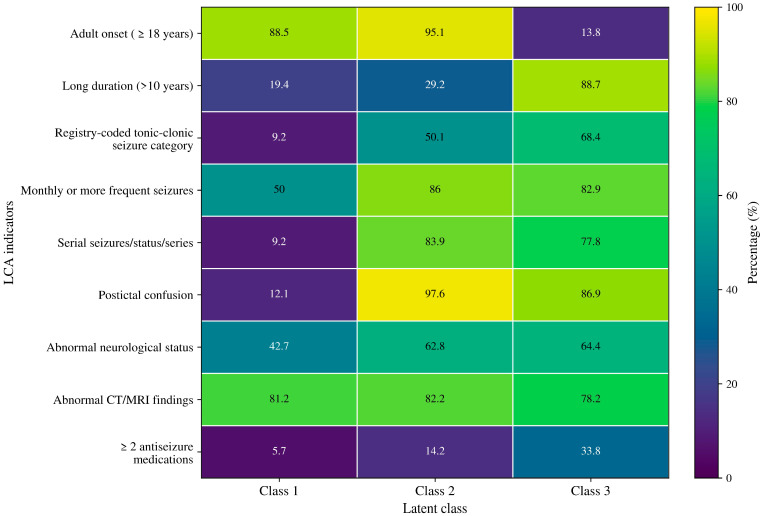
Latent class profile heatmap of predefined core LCA indicators across the three latent classes. Abbreviations: LCA, latent class analysis; CT, computed tomography; MRI, magnetic resonance imaging.

The final three-class solution identified clinically interpretable phenotypic profiles. Class 1 represented a lower-burden phenotype, characterized by lower seizure burden, less frequent serial seizures or status epilepticus, and lower treatment exposure. Class 2 represented an intermediate-burden phenotype with higher seizure activity and greater treatment exposure than Class 1. Class 3 represented the highest-burden phenotype, characterized by earlier onset, longer disease duration, more frequent adverse seizure-related features, and the highest proportion of DRE. The class-specific distribution of core LCA indicators is visualized in Figure 2.

DRE prevalence increased stepwise across the three latent classes. DRE was present in 18/314 patients in Class 1 (5.7%), 66/465 patients in Class 2 (14.2%), and 91/275 patients in Class 3 (33.1%). The between-class difference was statistically significant (χ^2^ = 82.62, *p* < 0.001), showing a stepwise gradient in DRE prevalence across the phenotypic profiles.

To improve the clinical interpretability of the three data-driven phenotypes, we performed a post hoc characterization according to epilepsy etiology, derived epilepsy type, and ILAE-aligned seizure categories (Table 1). Class 1 was predominantly composed of patients with a focal seizure phenotype (95.5%), especially focal impaired awareness seizures (54.8%) and focal aware seizures (35.7%), with structural/acquired or developmental etiologies in 62.7%. Class 2 also showed a predominantly focal seizure phenotype (80.0%), but with a higher proportion of focal to bilateral tonic–clonic seizures (30.5%) and structural/acquired or developmental etiologies (67.5%). Class 3 had the highest proportion of generalized/bilateral seizure phenotype (47.6%) and generalized/bilateral tonic–clonic seizures (45.1%), together with the highest proportion of unknown or not established etiology (52.0%). Thus, the latent classes differed not only by disease burden and observed DRE prevalence, but also by clinically meaningful differences in seizure-category and etiology composition.

**Description of latent classes.** Class 1 was interpreted as a lower-burden, predominantly adult-onset phenotype with lower seizure burden and treatment exposure. Class 2 represented an intermediate-burden phenotype characterized by high seizure activity despite predominantly adult onset. Class 3 represented an earlier-onset, long-duration, high-burden phenotype with the highest DRE prevalence and greater treatment exposure. Structural imaging abnormalities were frequent across all three classes.

**Internal clinical validation.** The latent classes also differed across multiple internal clinical validation outcomes that were not used as core LCA indicators. Median QOLIE-31 scores declined across classes from 70.0 (60.0–70.0) in Class 1 to 60.0 (40.0–70.0) in Class 2 and 52.0 (40.0–70.0) in Class 3 (statistic 87.473, *p* < 0.001). Median MoCA scores decreased from 22.0 (21.0–24.0) to 21.0 (18.0–22.0) and 20.0 (18.0–22.0), respectively (statistic 79.535, *p* < 0.001). MMSE showed a similar between-class difference, with medians of 23.0 (22.0–25.0), 22.0 (19.0–23.0), and 22.0 (19.0–23.0) (statistic 72.246, *p* < 0.001). Stigma scores were higher in the higher-burden classes, with medians of 37.5 (28.0–42.0), 41.0 (34.0–48.0), and 41.0 (32.0–46.0) (statistic 38.045, *p* < 0.001). ESS differed more modestly across classes, with medians of 22.0 (15.0–25.0), 22.0 (18.0–25.0), and 23.0 (19.0–25.0) (statistic 6.469, *p* = 0.039) (Table 2).

**Figure 3:**
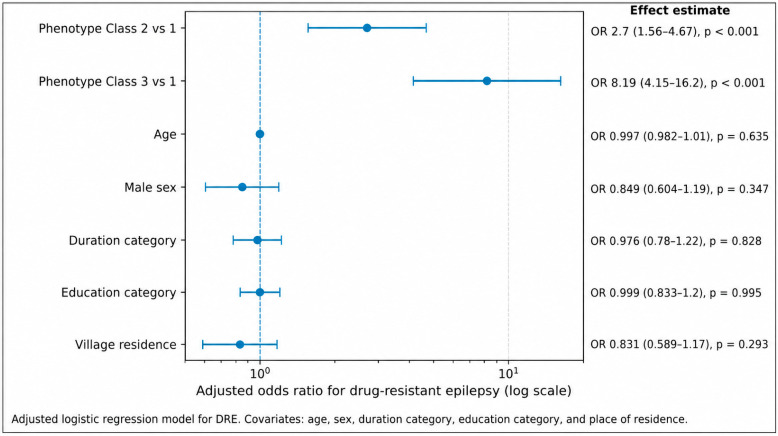
Forest plot of adjusted odds ratios for DRE. Blue circles indicate adjusted odds ratio point estimates, and horizontal lines represent 95% confidence intervals. The vertical dashed blue line indicates OR = 1. The model was adjusted for age, sex, duration category, education category, and place of residence.

Adjusted logistic regression. In adjusted logistic regression, latent class membership remained strongly associated with DRE after adjustment for age, sex, disease duration, education, and place of residence. Compared with Class 1, Class 2 had higher odds of DRE (OR 2.70, 95% CI 1.56–4.67), while Class 3 showed the strongest association (OR 8.19, 95% CI 4.15–16.16; both *p* < 0.001). None of the adjustment covariates materially attenuated the association between class membership and DRE. The adjusted model showed moderate discrimination, with an area under the receiver operating characteristic curve of 0.707 (Figure 3).

To address the potential circularity introduced by including the number of antiseizure medications as an LCA indicator while evaluating DRE as an outcome, we repeated LCA after excluding medication count. The alternative model showed substantial agreement with the primary classification, with an adjusted Rand index of 0.897. This finding suggests that the primary class structure was not driven solely by medication count, but reflected broader clinical patterns across seizure characteristics, disease duration, neurological status, and imaging category.

Model quality. The phenotype-based regression model showed an area under the curve of 0.707. Hosmer–Lemeshow chi-square was 8.263 with 8 degrees of freedom (*p* = 0.408), indicating acceptable calibration in the reported model. Variance inflation factors ranged from 1.009 to 2.367, without evidence of major multicollinearity. The comparator clinical variables model, reported without direct inclusion of antiseizure medication count and using ordinal coding, had a lower area under the curve of 0.682.

Sensitivity analysis. In sensitivity analysis excluding medication count from the LCA indicators, the 3-class structure remained the preferred solution by BIC. In that sensitivity model, AIC was 19,288.274, BIC 19,804.150, adjusted BIC 19,473.829, and entropy 0.793. Agreement between the primary and sensitivity class solutions was high, with an adjusted Rand index of 0.897, supporting the structural robustness of the phenotype solution beyond medication count alone. In the sensitivity-derived three-class solution excluding medication count, class sizes were 315, 484, and 255 patients, and DRE prevalence retained a stepwise gradient across classes (8.3%, 15.9%, and 28.2%, respectively). In adjusted logistic regression using the same covariates as the primary model, the association with DRE remained directionally consistent: Class 2 versus Class 1, OR 1.93, 95% CI 1.20–3.11; Class 3 versus Class 1, OR 2.46, 95% CI 1.32–4.58.

**Exploratory clinical framework.** The report-derived exploratory framework summarized the latent classes according to observed DRE prevalence and possible clinical considerations, rather than defining validated risk categories. Class 1 showed a low observed DRE burden, with DRE prevalence of 5.7%, and was linked to routine follow-up with monitoring of seizure control and adherence. Class 2 showed a moderate observed DRE burden, with DRE prevalence of 14.2%, and was linked to review of seizure burden, MRI status, comorbidities, and consideration of VEEG when clinically indicated. Class 3 showed a high observed DRE burden, with DRE prevalence of 33.1%, and was linked to explicit DRE assessment and consideration of epileptologist referral or multidisciplinary management. This framework should be interpreted as an exploratory conceptual summary rather than a validated clinical decision rule or prospective risk-prediction model (Figure S1).

## 4. Discussion

In this regional cohort of adults with epilepsy, LCA identified three clinically interpretable phenotypic profiles that differed in DRE prevalence and broader measures of disease burden. The main finding was a stepwise increase in DRE across the three classes, accompanied by lower quality of life, lower cognitive screening scores, and higher stigma scores in the higher-burden phenotypic profiles. These findings suggest that adult epilepsy in this cohort is better described by multidimensional clinical patterns than by isolated clinical variables alone. However, because the present study used a cross-sectional analytical framework, the identified classes should be interpreted as burden-enriched phenotypic profiles rather than as a validated prospective prediction tool.

The class structure was clinically coherent. Class 1 represented a lower-burden phenotype, whereas Class 3 was characterized by earlier onset, longer disease duration, more complex seizure burden, more frequent postictal symptoms, greater treatment exposure, and the highest prevalence of DRE. This pattern is consistent with previous evidence showing that treatment resistance in epilepsy is associated with repeated medication failure, higher seizure burden, structural abnormalities, neurological deficits, earlier onset, and more complex treatment histories [6,7,8,9,10]. In the present study, these factors did not appear as isolated predictors only; rather, they clustered into interpretable patient profiles. This is an important distinction because the latent class approach summarizes how several clinically relevant features co-occur within the same patient subgroup.

The post hoc clinical characterization provides an additional epileptological interpretation of the latent classes. Importantly, the classes should not be interpreted as distinct epilepsy syndromes or as replacements for formal ILAE classification. Rather, they represent data-driven clinical burden phenotypes with different compositions of seizure categories and etiological backgrounds. Class 1 was mainly a lower-burden focal seizure phenotype, Class 2 represented an intermediate-burden group with a substantial proportion of focal to bilateral tonic–clonic seizures and structural/acquired etiologies, whereas Class 3 combined earlier onset, long disease duration, higher DRE burden, and a larger proportion of generalized/bilateral tonic–clonic seizure coding and unknown etiology. This additional characterization helps clarify who these three groups are clinically and provides more region-specific information about the adult epilepsy population in Southern Kazakhstan.

The association between latent class membership and DRE was strong and graded. Compared with Class 1, Class 2 and especially Class 3 showed higher odds of DRE after adjustment for age, sex, disease duration, education, and place of residence. This suggests that the identified phenotypic profiles captured clinically meaningful differences in disease burden beyond basic demographic characteristics. The model showed moderate discrimination, supporting the interpretation of the classes as cross-sectional phenotypic profiles rather than a high-precision predictive algorithm.

The higher-burden latent classes were not defined by DRE alone, but were also associated with worse QOLIE-31 scores, lower MoCA and MMSE scores, and higher stigma scores. These findings support the interpretation of the classes as multidimensional burden profiles rather than seizure-control categories alone. They do not replace syndrome-level diagnosis or individualized treatment planning, as selected highly drug-resistant epilepsy cases may require specific therapeutic approaches [31].

The present analysis also adds a multidimensional layer to the interpretation of adult epilepsy in this regional cohort. Previous regional evidence has shown that medication adherence, seizure severity, and quality of life are interrelated dimensions of epilepsy burden [30]. The present study extends this perspective by evaluating how seizure-related, neurological, imaging, and treatment-related variables cluster together into latent phenotypic profiles. Thus, the contribution of the current analysis is not simply to confirm associations between individual outcomes, but to describe clinically interpretable patterns of disease burden that may help organize heterogeneity within the adult epilepsy population.

These findings may be particularly relevant for regional healthcare settings in which access to specialist expertise and advanced diagnostic pathways is unevenly distributed. In such contexts, structured phenotyping may help organize clinical information and highlight patients who may benefit from more detailed clinical review. However, whether this approach can improve referral pathways, follow-up strategies, or patient outcomes requires prospective evaluation. This interpretation is consistent with previous regional work supporting the feasibility and clinical value of structured phenotyping [32].

A potential methodological concern is the inclusion of medication count as an LCA indicator, because DRE is partly defined by failure of antiseizure medication regimens [5]. Sensitivity analysis excluding medication count showed a similar three-class structure and high adjusted Rand agreement, suggesting that the phenotype structure was not driven solely by treatment burden. However, partial circularity between treatment exposure and DRE cannot be fully excluded, and the magnitude of association between class membership and DRE should be interpreted cautiously. DRE is also a heterogeneous clinical category, and the present LCA-derived classes should not be interpreted as substitutes for syndrome-level diagnosis or individualized therapeutic decision-making. Overall, the findings support the value of data-driven phenotyping for describing clinical heterogeneity in adult epilepsy, while remaining hypothesis-generating.

### Limitations

This study has several limitations. First, the cross-sectional analytical framework does not allow causal inference or prospective prediction; therefore, the identified classes should be interpreted as phenotypic profiles associated with current disease burden. Second, the study was based on a single regional cohort from Southern Kazakhstan, and external validation in independent cohorts is required. Third, LCA was performed using complete cases; although the excluded proportion was relatively small, missing or invalid indicator values may have introduced selection bias. Fourth, medication count was included as an LCA indicator while DRE was used as an internal clinical validation outcome, creating a potential risk of partial circularity; however, sensitivity analysis excluding medication count supported the robustness of the class structure. Fifth, the class assignment was based on the most likely posterior probability, and some uncertainty in class membership is inherent to LCA. Finally, detailed syndrome-level epilepsy classification was not consistently available across the registry. Therefore, the post hoc clinical characterization should be interpreted as a descriptive summary based on available seizure-category, epilepsy-type, and etiology variables, rather than as a formal ILAE syndrome-level classification.

## 5. Conclusions

In this regional adult epilepsy cohort, LCA identified three clinically interpretable phenotypic profiles associated with a stepwise gradient in DRE and broader multidimensional disease burden. These findings support the value of data-driven phenotyping for describing clinical heterogeneity in adult epilepsy, but remain hypothesis-generating and require external and prospective validation before clinical use.

## Figures and Tables

**Table 1:** Post hoc clinical characterization of latent classes by epilepsy type, etiology, and ILAE-aligned seizure categories.

Variable	Class 1	Class 2	Class 3	Overall	*p*-Value
Focal seizure phenotype	300 (95.5)	372 (80.0)	144 (52.4)	816 (77.4)	<0.001
Generalized/bilateral seizure phenotype	14 (4.5)	93 (20.0)	131 (47.6)	238 (22.6)	-
Focal aware seizure	112 (35.7)	0 (0.0)	7 (2.5)	119 (11.3)	<0.001
Focal impaired awareness seizure	172 (54.8)	230 (49.5)	73 (26.5)	475 (45.1)	-
Focal to bilateral tonic–clonic seizure	16 (5.1)	142 (30.5)	64 (23.3)	222 (21.1)	-
Generalized/bilateral tonic–clonic seizure	13 (4.1)	91 (19.6)	124 (45.1)	228 (21.6)	-
Unknown/not established etiology	112 (35.7)	139 (29.9)	143 (52.0)	394 (37.4)	<0.001
Structural/acquired/developmental etiology	197 (62.7)	314 (67.5)	118 (42.9)	629 (59.7)	-
Infectious etiology	5 (1.6)	10 (2.2)	14 (5.1)	29 (2.8)	-

**Table 2:** Internal clinical validation outcomes across latent classes.

Outcome	Class 1	Class 2	Class 3	Statistic	*p*-Value
DRE, *n*/N (%)	18/314 (5.7)	66/465 (14.2)	91/275 (33.1)	χ^2^ = 82.62	<0.001
QOLIE-31, median (IQR)	70.0 (60.0–70.0)	60.0 (40.0–70.0)	52.0 (40.0–70.0)	87.5	<0.001
MoCA, median (IQR)	22.0 (21.0–24.0)	21.0 (18.0–22.0)	20.0 (18.0–22.0)	79.5	<0.001
MMSE, median (IQR)	23.0 (22.0–25.0)	22.0 (19.0–23.0)	22.0 (19.0–23.0)	72.2	<0.001
ESS, median (IQR)	22.0 (15.0–25.0)	22.0 (18.0–25.0)	23.0 (19.0–25.0)	6.47	0.039
Stigma score, median (IQR)	37.5 (28.0–42.0)	41.0 (34.0–48.0)	41.0 (32.0–46.0)	38.0	<0.001

## Data Availability

The raw data supporting the conclusions of this article will be made available by the corresponding author on request.

## Supplementary Materials

The following supporting information can be downloaded at: https://www.mdpi.com/article/10.3390/jpm16070344/s1, Table S1. Variable-specific missingness and invalid or non-informative codes across core LCA indicators; Table S2. Baseline clinical and demographic characteristics of the adult analytic cohort; Table S3. Latent class model fit indices and class distribution; Figure S1. Exploratory conceptual framework based on latent phenotypic profiles.

## Abbreviations

The following abbreviations are used in this manuscript:

ASMs	antiseizure medications
BIC	Bayesian information criterion
CT	computed tomography
DRE	drug-resistant epilepsy
ESS	Epilepsy Stigma Scale
ILAE	International League Against Epilepsy
IQR	interquartile range
LCA	latent class analysis
MMSE	Mini-Mental State Examination
MoCA	Montreal Cognitive Assessment
MRI	magnetic resonance imaging
QOLIE-31	Quality of Life in Epilepsy Inventory-31
VEEG	video-electroencephalography
