# Supplementary material for: Data-Driven Clinical Phenotyping of Adult Epilepsy Using Latent Class Analysis: A Regional Cohort Study from Southern Kazakhstan
Source: J Pers Med. 2026 Jun 25;16(7):344. doi: 10.3390/jpm16070344 (PMC13413036; doi:10.3390/jpm16070344)
Supplement: Supplementary file 1 [file jpm-16-00344-s001.zip › Table S1. Variable-specific missingness and invalid or non-informative codes across core LCA indicators.pdf]

Table S1. Variable-specific missingness and invalid/non-informative codes across core LCA indicators. Percentages were calculated using the corrected adult analytic cohort as the denominator (n = 1,098). Non-informative or invalid codes, including "unknown," "not examined," or invalid category values, were recoded as missing. Because some patients had more than one affected indicator, the sum of variable-level counts does not equal the number of excluded records. Overall, 44 records were excluded from the complete-case LCA sample, resulting in a final LCA sample of 1,054 patients.

**Core LCA indicator**	**Missing/unknown/not examined/invalid, n**	**Percentage of adult analytic cohort, %**
Age at epilepsy onset	2	0.2
Disease duration	5	0.5
Seizure type	0	0.0
Seizure frequency	0	0.0
Serial seizures/status epilepticus	39	3.6
Postictal confusion	0	0.0
Neurological status	1	0.1
CT/MRI category	1	0.1
Number of antiseizure medications	0	0.0
