# Supplementary material for: Data-Driven Clinical Phenotyping of Adult Epilepsy Using Latent Class Analysis: A Regional Cohort Study from Southern Kazakhstan
Source: J Pers Med. 2026 Jun 25;16(7):344. doi: 10.3390/jpm16070344 (PMC13413036; doi:10.3390/jpm16070344)
Supplement: Supplementary file 1 [file jpm-16-00344-s001.zip › Table S2. Baseline clinical and demographic characteristics of the adult analytic cohort.pdf]

**Table S2. Baseline clinical and demographic characteristics of the adult analytic cohort.** Values are presented as mean ± standard deviation and median with interquartile range for continuous variables, and as number and percentage for categorical variables. The corrected adult analytic cohort included 1,098 patients after exclusion of two records aged <18 years. MoCA, Montreal Cognitive Assessment; MMSE, Mini-Mental State Examination; ESS, Epilepsy Stigma Scale; QOLIE-31, Quality of Life in Epilepsy Inventory-31.

| Variable | n | Mean ± SD | Median (IQR) | Min–Max |
|---|---|---|---|---|
| Age, years | 1,098 | 40.5 ± 14.3 | 39 (29–51) | 18–87 |
| MoCA score | 1,098 | 20.5 ± 3.89 | 21 (18–23) | 7–28 |
| MMSE score | 1,097 | 21.5 ± 3.93 | 22 (19–25) | 7–30 |
| ESS score | 1,098 | 21.4 ± 5.02 | 23 (18–25) | 10–32 |
| Stigma score | 1,098 | 38.2 ± 10.5 | 40 (31–45) | 10–65 |
| QOLIE-31 score | 1,098 | 57.5 ± 15.5 | 60 (45–70) | 10–90 |
