# Supplementary material for: Data-Driven Clinical Phenotyping of Adult Epilepsy Using Latent Class Analysis: A Regional Cohort Study from Southern Kazakhstan
Source: J Pers Med. 2026 Jun 25;16(7):344. doi: 10.3390/jpm16070344 (PMC13413036; doi:10.3390/jpm16070344)
Supplement: Supplementary file 1 [file jpm-16-00344-s001.zip › Table S3. Latent class model fit indices and class distribution.pdf]

Table S3. Latent class model fit indices and class distribution. LCA was performed in the corrected adult complete-case sample after exclusion of records with invalid or missing LCA indicators. Model selection was based on BIC, entropy, minimum class size, class distribution, and clinical interpretability. The 3-class solution was selected because it had the lowest BIC, acceptable entropy, and clinically interpretable class sizes. AIC, Akaike information criterion; BIC, Bayesian information criterion; LCA, latent class analysis.

Class №	n	Log-likelihood	AIC	BIC	Adjusted BIC	Entropy	Min. class size	Class sizes	Selected model
2	1,054	-1.02×10^4	2.05×10^4	2.09×10^4	2.07×10^4	0.812	353	701, 353	No
3	1,054	-1.00×10^4	2.03×10^4	2.08×10^4	2.05×10^4	0.791	275	275, 314, 465	Yes
4	1,054	-9.92×10^3	2.01×10^4	2.09×10^4	2.04×10^4	0.810	155	164, 235, 500, 155	No
5	1,054	-9.82×10^3	2.00×10^4	2.09×10^4	2.03×10^4	0.806	105	105, 403, 156, 221, 169	No
