# Supplementary figures and images for: Data-Driven Clinical Phenotyping of Adult Epilepsy Using Latent Class Analysis: A Regional Cohort Study from Southern Kazakhstan
Source: J Pers Med. 2026 Jun 25;16(7):344. doi: 10.3390/jpm16070344 (PMC13413036; doi:10.3390/jpm16070344)

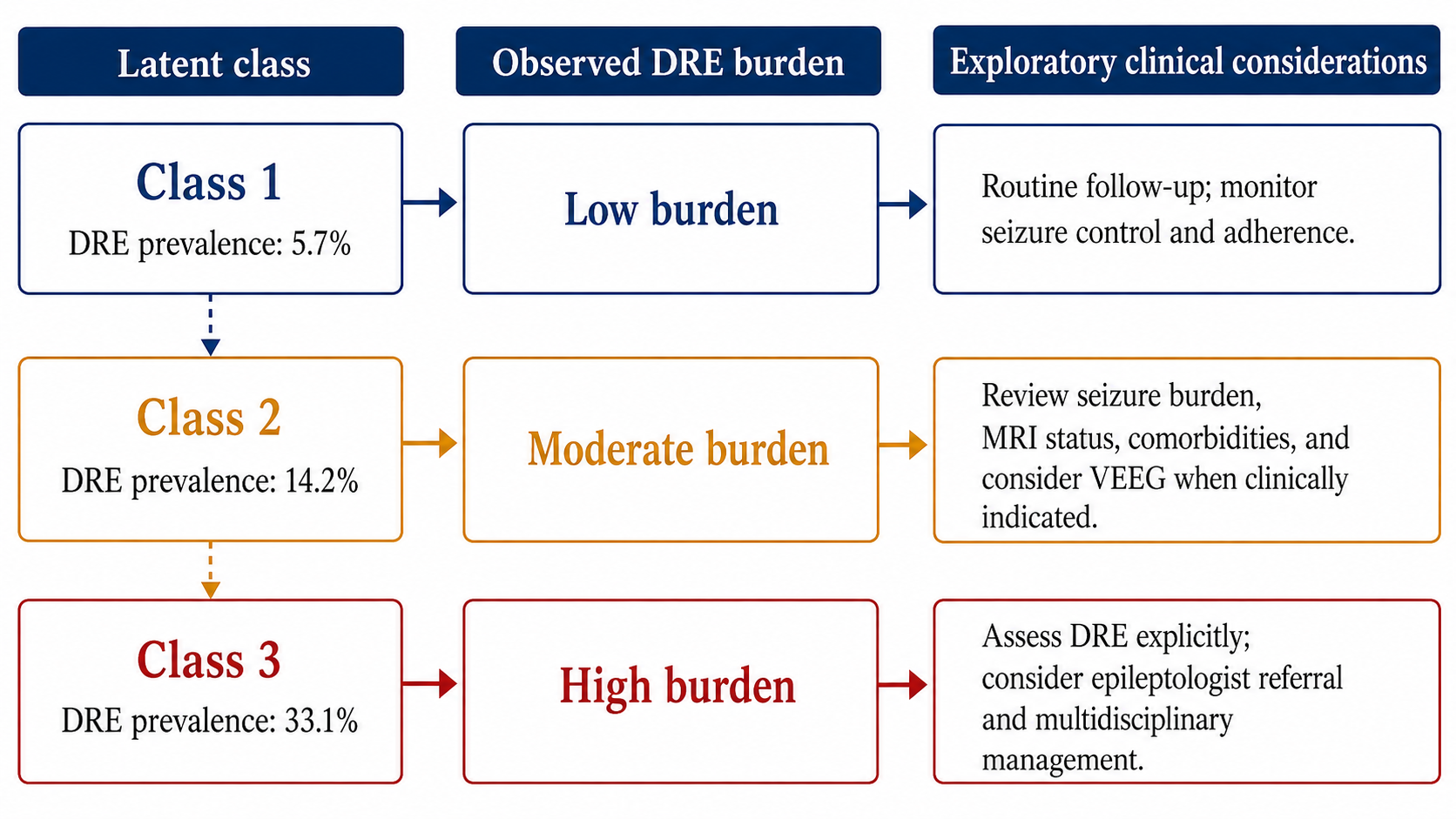

Supplement: Supplementary file 1 [file jpm-16-00344-s001.zip › Figure S1. Exploratory conceptual framework based on latent phenotypic profiles..png]
